# Network analysis of the NetHealth data: exploring co-evolution of individuals’ social network positions and physical activities

**DOI:** 10.1007/s41109-018-0103-2

**Published:** 2018-11-02

**Authors:** Shikang Liu, David Hachen, Omar Lizardo, Christian Poellabauer, Aaron Striegel, Tijana Milenković

**Affiliations:** 1 Department of Computer Science and Engineering, University of Notre Dame, Notre Dame, 46556 IN USA; 2 Department of Sociology, University of Notre Dame, Notre Dame, 46556 IN USA; 3 Eck Institute for Global Health, University of Notre Dame, Notre Dame, 46556 IN USA; 4 Interdisciplinary Center for Network Science and Applications (iCeNSA), University of Notre Dame, Notre Dame, 46556 IN USA

**Keywords:** Dynamic networks, Social networks, Node centrality, Health, Physical activity

## Abstract

Understanding the relationship between individuals’ social networks and health could help devise public health interventions for reducing incidence of unhealthy behaviors or increasing prevalence of healthy ones. In this context, we explore the co-evolution of individuals’ social network positions and physical activities. We are able to do so because the NetHealth study at the University of Notre Dame has generated both high-resolution longitudinal social network (e.g., SMS) data and high-resolution longitudinal health-related behavioral (e.g., Fitbit physical activity) data. We examine trait differences between (i) users whose social network positions (i.e., centralities) change over time versus those whose centralities remain stable, (ii) users whose Fitbit physical activities change over time versus those whose physical activities remain stable, and (iii) users whose centralities and their physical activities co-evolve, i.e., correlate with each other over time. We find that centralities of a majority of all nodes change with time. These users do not show any trait difference compared to time-stable users. However, if out of all users whose centralities change with time we focus on those whose physical activities also change with time, then the resulting users are more likely to be introverted than time-stable users. Moreover, users whose centralities and physical activities both change with time and whose evolving centralities are significantly correlated (i.e., co-evolve) with evolving physical activities are more likely to be introverted as well as anxious compared to those users who are time-stable and do not have a co-evolution relationship. Our network analysis framework reveals several links between individuals’ social network structure, health-related behaviors, and the other (e.g., personality) traits. In the future, our study could lead to development of a predictive model of social network structure from behavioral/trait information and vice versa.

**Electronic supplementary material:**

The online version of this article (10.1007/s41109-018-0103-2) contains supplementary material, which is available to authorized users.

## Introduction

### Problem statement and related work

Individuals’ health is closely related to their social networks (Latkin and Knowlton 2015; Perkins et al. 2015; Pastor-Satorras et al. 2015; Valente and Pitts 2017; Cobb et al. 2010). Understanding this relationship could help devise network-based strategies to reduce the incidence of unhealthy behaviors or increase the prevalence of healthy ones.

The interplay between individuals’ social networks and traits (including health) has been extensively studied for decades (Borgatti et al. 2009; Smith and Christakis 2008). One research direction focuses on social ties and the trait (dis)similarity between interacting individuals (O’Malley and Christakis 2011; Valente et al. 2013; McPherson et al. 2001). For example, some studies analyzed whether individuals who have (dis)similar traits, such as genotype (Fowler et al. 2011), personality (Robins et al. 2000; Selfhout et al. 2010), race (Mollica et al. 2003), or others (Sepulvado et al. 2015), are likely to form (or dissolve) ties. An alternative avenue that we focus on in this paper explores how social network positions (centralities) of individuals are associated with their traits, which is important to study for reasons discussed below.

In this context, traditionally, due to the inability to collect longitudinal social interactions and trait data, scholars studied the relationship between network positions and traits cross-sectionally, at a single time point – in a static fashion. For example, it was shown that people with specific traits, such as obesity, tend to have different (higher or lower, depending on the trait) centrality values in a static social network (Strauss and Pollack 2003). As another example, individuals’ positions in a static social network were shown to be correlated with their personalities (Staiano et al. 2012; Klein et al. 2004), creativities (Gloor et al. 2011; Perry-Smith and Shalley 2003), and self-reported health (Youm et al. 2014).

However, social systems, just like most other complex real-world systems, evolve with time. The static representation of such a dynamic system clearly ignores valuable temporal information (Holme 2015). Luckily, more recently, longitudinal social interaction data collected from smartphones became accessible, which could be modeled as a dynamic network (Meng et al. 2014; Zhang et al. 2017). Yet, trait data remained static. In this context, scholars studied the interplay between individuals’ evolving network positions and their static traits. For example, we showed that clusters of individuals with similar evolving network centralities (e.g., according to phone call or Facebook interactions) correspond well to clusters of individuals with similar static traits (e.g., gender or agreeableness) (Meng et al. 2016).

At present, individuals’ longitudinal trait data can be collected as well, e.g., via Fitbit devices. This is what we have done in the NetHealth study, which monitors smartphone usage (e.g., SMS interactions) and health-related behaviors (e.g., physical activities, heart rates, or sleep habits) of ∼700 Notre Dame undergraduates over more than two years (Purta et al. 2016; Faust et al. 2017). With such data, we are able to study the relationship between individuals’ centralities in *dynamic* networks and their *dynamic* health-related behaviors, with focus on physical activity behaviors in this study. More specifically, we deal with the following problem: We are able to look at the co-evolution of a person’s network position (considering a variety of centrality measures) and their behavior (i.e., physical activity), with the goal of identifying groups of people who have different evolving social network profiles or different evolving physical activity profiles (or both). We are then able to explore how the different individuals differ in terms of personality, depression, and anxiety traits, as a preliminary step towards understanding how and when network position and behaviors (i.e., physical activity) change together.

Note that there exist studies that used longitudinal smartphone or wearable sensor data to infer users’ static traits, such as personality (Chittaranjan et al. 2013; Chittaranjan et al. 2011; Golbeck et al. 2011), sleep quality (Sano et al. 2015), and academic performance (Sano et al. 2015; Wang et al. 2014) or longitudinal traits, such as health status (Madan et al. 2010) and stress (Bogomolov et al. 2014). However: 1) Instead of constructing a network of the individuals’ social interactions, these studies focused on individual-level features, such as the average duration of calls, number of missed calls, Internet usage, or number of contacts (Aharony et al. 2011), and were thus not network-based like our study. 2) Because they were not network-based, they were unable to study the relationship between individuals’ social networks and their traits, which is what we focus on in this paper.

Also, there exist some studies of longitudinal social interaction data and longitudinal health-related trait data that *are* network-based, just like our study is (Christakis and Fowler 2007; Christakis and Fowler 2008; Fowler and Christakis 2008; Cohen-Cole and Fletcher 2008). However, such studies mainly focused on dyadic (person-to-person) diffusion, rather than on looking at the connection between network centrality and health outcomes like we do. This could be because the previous studies collected data via surveys, which suffer from long time span (often years), yet limited time points. Also, these studies’ ego-networks were capped at only several alters. Moreover, social networks mapped by surveys, including those of the existing studies, change little with time, because most of links are relatives and close friends. In other words, these studies did not have enough data or data of an appropriate type to be able to perform our analyses and ask the questions that we are asking in this paper. Compared to surveys, our continuous electronic (cell phone and Fitbit) records provide high-validity, fine-grained, temporal data. This allows us to not only study dyadic diffusion from one time point to another (which is out of the scope of this paper), but also to analyze evolving patterns of individuals’ network positions (centralities) and their health-related behaviors (physical activities). We believe that focusing on the notion of network centrality rather than on dyadic interactions may be a more promising avenue in terms of coming up with practical interventions, as the former captures much richer network topological information. Yet, despite this promise, to our knowledge, there has been little work looking at the effects of individual network position on health outcomes, especially using a variety of measures of centrality.

In fact, the only existing studies that we are aware of that perform longitudinal “network position versus trait” data analyses deal with other, non-health-related applications. For example, temporal Bollywood actor network data, including their multilayer version, were analyzed to differentiate successful actors from the others by studying the actors’ local network properties at different times (Sarkar et al. 2016; Jalan et al. 2014).

### Our study and contributions

**Fig. 1:**
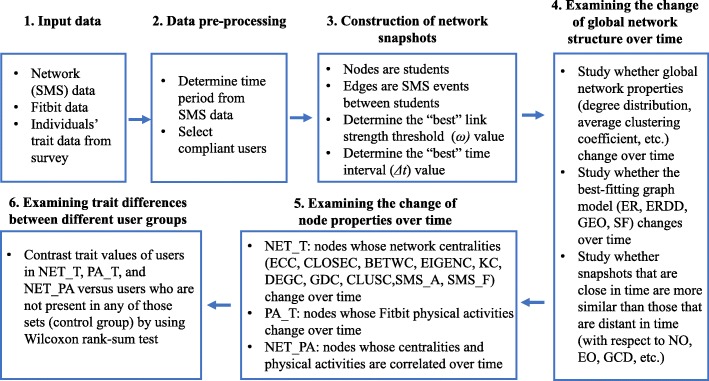
Summary of our study

The summary of our study is as follows (Fig. 1):

- We identify compliant users with reliable social interaction (SMS) data within our 12-month study period. We construct a dynamic network of SMS interactions between 576 such users, by considering weekly network snapshots.
- We study the evolution of the dynamic network, i.e., its global as well as local network structure, with time.
- We examine whether individuals with certain behaviors in terms of evolving social network positions (i.e., centralities) or physical activities have certain (personality, depression, anxiety, etc.) traits.
- In terms of network evolution, we see low similarity between school week snapshots and break (holiday) week snapshots. Focusing only on the more meaningful school week snapshots, we see high node overlaps. However, only snapshots from consecutive time periods have high edge overlaps. Yet, even though many edges form and break with time, global network properties such as the snapshots’ degree distributions remain stable.
- On the other hand, the local centralities of 74% of all nodes significantly change with time. These users do not show any trait difference compared to time-stable users. However, if out of all the users whose centralities change with time we focus on those whose physical activities also change with time, then the resulting (21% of all) users are more likely to be introverted than time-stable users. Moreover, evolving centralities are significantly correlated (i.e., co-evolve) with evolving physical activities for 27% of all nodes. Users whose centralities and physical activities both change with time and who also have a co-evolution relationship (12% of all users) are more likely to be introverted as well as anxious compared to those users who are time-stable and do not have a co-evolution relationship.
- Hence, our study reveals interesting links between individuals’ social network structure, health-related behaviors such as physical activity, and other (e.g., personality) traits.

## Methods

### Data description

#### Overview

Our data come from the NetHealth study, an institutional review board-approved ongoing effort to collect phone, Fitbit, and survey data from approximately 700 undergraduate student volunteers at the University of Notre Dame, who entered the study as freshmen in August 2015. Phone data is collected via a monitoring application installed on participants’ smartphones that periodically synchronizes text message logs and other data (Hossain and Poellabauer 2016). Health behavioral data is collected via Fitbit Charge HR devices. Online surveys are conducted once a semester to ascertain the views of the study participants on a wide range of subjects.

#### SMS data

In this study, we use SMS logs to map individuals’ social networks. We do not collect information about the content of the messages. Instead, we collect information on who communicates with whom and when. Each SMS log is associated with the two individuals involved in the communication (sender and receiver) and a time stamp.

#### Fitbit data

Students’ health behavioral data are collected via Fitbit Charge HR devices provided by the NetHealth study. Fitbit metrics consist of three main categories: 1) physical activities such as step counts, floors climbed, and calories burned, 2) sleep habits such as bed time and sleep duration, and 3) heart rate. In this study, we mainly focus on physical activities, in particular, the participants’ step counts.

In addition, we measure users’ Fitbit compliance based on heart rate information (Purta et al. 2016; Faust et al. 2017). The daily compliance rate is calculated as the number of minutes in a day during which the Fitbit device detects a heart rate divided by the total number of minutes in a day (1440).

#### Individuals’ static trait data from surveys

We conduct periodic surveys that ask information about participants’ demographics, prior education, personality traits, mental health, sleep quality, political affiliations, etc. In this study, we extract seven traits from the survey describing a person’s mental health conditions and personality, namely depression, anxiety, agreeableness, extroversion, openness, conscientiousness, and neuroticism. Depression and anxiety levels are measured by the Center for Epidemiological Studies Depression Scale (CESD) test and the State-Trait Anxiety Inventory (STAI) test, respectively. Individuals’ personalities are measured by the Big-Five personality test. We extract trait values from the survey taken in the spring of 2016 (specifically, in January 2016). This is because this survey was conducted during the August 2015 – August 2016 period that we consider in this paper (for reasons explained below), which corresponds to year one of the NetHealth study. Since we deal with only one survey time point (wave), we treat the above seven traits as static. However, note that we collect the same survey data at additional waves, in each semester of years two and three of the NetHeatlh study. So, in theory, some participants’ personalities might change over the three years, although we do not expect major changes during this still reasonably short time period (e.g., compared to one’s entire life span). To illustrate this, we compute the correlation coefficients of participants’ personality traits across the different waves, which are relatively high, in the 0.65-0.85 range.

### Data processing

Real-world data contain missing values and irrelevant information. To deal with these issues, we clean up the raw NetHealth dataset before constructing a network from it. This section describes how we determine the time period of our study and select compliant users.

#### Determining the time period and user pool from SMS data

The NetHealth database contains SMS logs of 615 iPhone users and 96 Android users. In this study, we only consider iPhone data because it has better quality than Android data due to Android agent implementation issues. Note that the NetHealth study collects not only its participants’ SMS data but indirectly also SMS data of whoever the participants interact with outside of the study. In this paper, we discard all SMS data to/from the outsiders, and we only keep SMS data between the 615 within-study iPhone users.

The problems of selecting the time period and selecting the pool of users are interdependent. As time goes on, more and more users either drop from the study or become non-compliant. Hence, selecting a longer time period would lead to a smaller user pool. However, selecting a shorter time period would encompass less temporal information about users’ SMS and health-related behaviors. Thus, we need to find a balance between the length of the time period and the size of the user pool, which we do as follows. We say that a user is active if they send or receive an SMS. From the SMS data, we extract information about each user’s first activity date and last activity date, i.e., the user’s “active time interval”. Then, for a given time period *T* during the study, we examine: 1) how many users are active during time period *T* (i.e., the users’ active time intervals “cover” the entire time period *T*), 2) how many users are active in the given week of *T*, for each week, and 3) how many users have dropped from the study prior to the end of time period *T*. We want to select a time period *T* such that items 1 and 2 are satisfied by as many users as possible, while item 3 is satisfied by as few users as possible.

We observe that during the first year of the NetHealth study: 1) 576 of the 615 users are actively involved in the study, 2) 550 of the 615 users are active per school week on average over all school weeks, and 431 of the 615 users are active per break week on average over all break weeks, and 3) only 17 users have dropped from the study prior to the end of the first year (Additional file 1: Figure S1). Thus, the first year period meets our above criteria. Also, we started the work in this paper close to the end of the second year of the study. Consequently, the data for the second year was incomplete. Therefore, in this paper, we select the first year period from August 24, 2015 (the first day of the fall 2015 semester) to August 22, 2016 (the first day of the fall 2016 semester) as the period of interest. This period covers 52 weeks, of which 31 are school weeks and 21 are break weeks.

We filter out users who formally dropped out of the study or became completely inactive (i.e., did not send or receive any SMSs) within our study period, because SMS data for such users is incomplete and could introduce biases into our subsequent analyses of the dynamic network. This leaves us with 576 study participants as our user pool for constructing the network. We denote this pool of users as NetU. These users have sent or received around three million SMSs during our study period. For more details on numbers of weeks in which NetU users are active, see Additional file 1: Section S1 and Figure S2.

#### Determining the user pool when studying co-evolution of social networks and physical activities

We construct a dynamic network of SMS interactions between the users from the NetU set, who we ensured are compliant in terms of SMS data, per our above discussion. When examining the co-evolution relationship between users’ network positions and Fitbit physical activities, we consider a subset of the users in NetU that are also compliant in terms of Fitbit data. We identify such users as follows.

Two main challenges with Fitbit data are both related to missing data, as follows. First, some people do not sync their data to Fitbit (either by using the Fitbit App or the dongle that comes with the Fitbit) in time. Since the Fitbit Charge HR device can only hold about seven days worth of (detailed minute-by-minute data) data, if not caught early, syncing issues can result in data loss. Second, some users have low compliance rates in certain days, meaning that they do not wear their Fitbit devices enough time in a given day.

We deal with these two challenges as follows. The Fitbit dataset contains daily step counts and compliance rates of 520 of the 576 NetU users. From these 520 users’ data, first, we remove daily records that have compliance rates below 80% (Purta et al. 2016; Faust et al. 2017). Then, to be consistent with our dynamic network analyses, where in the dynamic network we aggregate *weekly* SMS events (for reasons discussed in “Network construction” section), we also “bin” daily Fitbit step counts into weekly step counts by taking the average of step counts over all days in the given week. For a given user, if no step count value is found in a given week, we mark it as missing. We filter out users whose step counts are missing for at least half of all weeks of the first year time period. After the filtering, 302 users from NetU remain as our user pool for detecting the co-evolution relationship. We denote this 302-user pool as CoU.

Note that we use NetU instead of CoU to construct the dynamic network and compute nodes’ social network positions (centralities), in order to include as much (reliable) information as possible into the network construction process and thus better characterize network positions of all users. Then, we use CoU and their network centrality values (computed based on their interactions with all NetU users) when studying co-evolution of the centralities and CoU’s physical activities.

### Network construction

Constructing a series of network snapshots of equal time interval length *Δt* is the most common way to model a dynamic network from longitudinal data (Zhang et al. 2017), which is what we also do in this paper. For each snapshot, nodes are the users in NetU and there is an edge between two nodes if there are at least *w* SMS events between the two nodes within the given time interval.

Different configurations of parameters *Δt* and *w* result in different networks. The choice of a small *Δt* or a large *w* could lead to largely disconnected network snapshots. On the other hand, the choice of a large *Δt* could lead to loss of valuable longitudinal information. Also, the choice of a large *Δt*, or the choice of a small *w*, could lead to highly dense network snapshots. Thus, we aim to find some intermediate *Δt* and *w* values that would generate meaningful network structures.

For this purpose, we measure the density and the size of the largest connected component (LCC) of networks resulting from different *Δt* and w choices. For graph G = (V, E), the density of G is $\frac {2|E|}{|V|\times |V-1|}$, and its LCC size is defined as the number of nodes in the LCC divided by |*V*|. Real-world networks are typically sparse and have large LCC sizes (Newman 2010). Hence, we want to find a balance between small network density and large LCC size. We vary *Δt* from 1 week to 6 months and we vary *w* for each *Δt* (Table 1). In this study, we choose *Δt*=1 week and *w*=1 to construct the network, because any larger *Δt* loses most of the temporal information, and for *Δt*=1, the choice of *w* did not have any major effect (during school weeks, which is what we focus on in most of our analyses, for reasons discussed later on in the manuscript). For more details, see Additional file 1: Section S2 and Figure S3.

**Table 1:** Values for time interval length *Δt* and edge threshold *w* that we evaluate in this paper

Time interval (*Δt*)	Link threshold (*w*)
1 week	1,2,3,4
1 month	1,2,3,4
3 months	1,3,6,9
1 semester	1,3,6,9
6 months	1,6,12,18

### Examining the change of global network structure over time

Given the dynamic network, we study how the network evolves over time. First, we test whether global topological properties of the network snapshots change with time. We do so by studying the evolution of (change in) global network properties of the different snapshots with time, by evaluating the fit of each of the network snapshots to a series of well-known network (random graph) models (Kuchaiev et al. 2011; Milenković et al. 2008), and by computing similarities between the different snapshots (Meng et al. 2014), as follows.

#### Studying the evolution of global properties of network snapshots

We analyze commonly used global network properties of each network snapshot, including the degree distribution, average clustering coefficient, clustering spectrum, average shortest path length, shortest path length distribution, and modularity (Kossinets and Watts 2006). These properties are defined in Additional file 1: Section S3. We compare the average clustering coefficient and the average shortest path length of the given network snapshot with the same properties of 30 random network instances drawn from the generalized Erdős–Rényi random graph model that preserves the degree distribution of the data (ERDD) (Milenković et al. 2008), where the random model networks have the same size as the snapshot in question. We compute *z*-scores to quantify the statistical significance of the given property value in the data network compared to the random networks.

#### Evaluating the fit of the dynamic network to different network models, i.e., graph families

For each network snapshot, we compare its fit to different network models (Milenković et al. 2009): 1) Erdős–Rényi random graphs (ER), 2) ERDD, 3) geometric random graphs (GEO), and 4) scale-free networks (SF) (Kuchaiev et al. 2011; Milenković et al. 2008). To evaluate the fit of a snapshot to a given model, we compare the topology of the snapshot with topologies of 30 random network instances drawn from the model with respect to a highly constraining measure of network topological similarity, called graphlet degree distribution agreement (GDDA).

#### Computing similarities between network snapshots at different time points

To study network evolution, we compare network snapshots from different time points. To compare any two network snapshots, we use the following network similarity measures: 1) node overlap (NO), the relative overlap of the networks’ nodes, as measured by Jaccard index (the size of the overlap of two sets divided by the size of the union of the two sets); 2) edge overlap (EO), the relative overlap of the networks’ edges, as measured by Jaccard index; 3) graphlet correlation distance (GCD) (Yaveroğlu et al. 2014), a sensitive measure of network topological similarity that is based on the notion of graphlets (subgraphs, small Lego-like building blocks of complex networks) (Milenković and Pržulj 2008); 4) GDDA, which generalizes the degree distribution of a network to its graphlet degree distributions (GDDs), one GDD for each of 73 2-5-node graphlets (Pržulj 2007); 5) relative graphlet frequency distance (RGFD), which compares the frequencies of the appearance of all 3–5-node graphlets in two networks (Milenković et al. 2008); and 6) Spearman correlation between local node properties (centralities) over all nodes (10 centrality measures that we consider are described in “Local network centralities” section).

For each measure, we form a snapshot similarity matrix *A*_*N*×*N*_ where N is the number of network snapshots (i.e., weeks in our data) and element *A*_*i*,*j*_ of *A*_*N*×*N*_ is the value of network similarity between the snapshots from weeks *i* and *j*. Note that the snapshot similarity matrix is symmetric. We study relationships between the different measures (NO, EO, GCD, GDDA, RGFD, and the 10 centrality measures) by comparing their corresponding snapshot similarity matrices, i.e., by computing Spearman correlations between them. We study potential redundancies of the different centrality measures by computing, for each pair of the measures, the Spearman correlation between their values over all nodes in a network snapshot, and by averaging the resulting correlations over all snapshots.

### Examining the change of local network structure (centralities) over time

#### Local network centralities

We measure social network positions of all nodes in each snapshot via 10 measures, each of which aims to capture the importance of a node in a network, often from a complementary perspective compared to others (Milenković et al. 2011; Faisal and Milenković 2014). The 10 measures are: eccentricity centrality (ECC), closeness centrality (CLOSEC), betweenness centrality (BETWC), eigenvector centrality (EIGENC), *k*-coreness centrality (KC), degree centrality (DEGC), graphlet degree centrality (GDC), clustering coefficient (CLUSC), SMS activity (SMS_A), and SMS frequency (SMS_F). SMS activity is defined as the number of days in a given week in which a user sent or received at least one SMS message. SMS frequency is defined as the number of SMS messages that a user sent or received in a given week. Thus, while SMS activity and SMS frequency are not explicit measures of network structure, they can be thought of as measures of the nodes’ “weighted degrees”, which is why we thus treat them as implicit network centrality measures. Definitions of the other, more traditional (explicit) network centrality measures are given in Additional file 1: Section S4.

#### Finding users whose network centralities evolve over time

Because global network analysis reveals that school weeks and break weeks have different network structures, with school weeks having more meaningful structures than break weeks (“Results” section), henceforth, we focus only on school weeks.

For each centrality measure, for each user in CoU, we compute the given user’s centrality values in all school week network snapshots. Then, we compute the Spearman correlation between the user’s centrality values and time. We test the significance of the correlation using cor.test function in R (Best and Roberts 1975). We compute this correlation for all users and find those whose centrality values are significantly correlated with time (*p*<0.01), with respect to *at least one* centrality measure; we use the “at least one” constraint because the different centrality measures may be complementary and could thus capture somewhat different sets of users. We denote the resulting set of users as NET_T.

Note that in a subsequent analysis, when we study both centrality values and physical activities, we need to remove for a given user their centrality value in a given week if their physical activity has a missing value in that week. To be consistent between the different analyses, here we also use only those weeks in which the given user has present both centrality values and physical activity values. In other words, when computing correlations, the weeks with missing values are ignored.

#### Statistical significance of the number of users whose network positions evolve over time

We study whether the number of users whose centralities significantly change with time (NET_T) is statistically significantly larger than the number of users who could be identified at random. By random, we mean randomly shuffling the time order of network snapshots. By doing this, we only change the time variable, but we keep each network snapshot the same. To take into account non-deterministic nature of the above randomization procedure, we repeat the procedure 100 times. Then, we compute *z*-scores to test the significance, as: *z*=(*x*−*μ*)/*σ*, where *x* is the observed number of users, *μ* is the average number of users over the 100 random procedure runs, and *σ* is the standard deviation associated with this average.

#### Relationships and potential redundancies of different node centralities

In order to test the redundancies of sets of users found by different centralities, we study: 1) the number of users who are found by exactly *k* (*k*=1,2,...10) centrality measures, 2) the number of users who are uniquely found by a given centrality measure, and 3) the overlap between users found by each pair of centrality measures. The overlap is measured by Jaccard index. We also test the statistical significance of the overlap by using the hypergeometric test, which computes the probability P (i.e., *p*-value) of observing the same or larger overlap by chance.

### Examining the change of users’ physical activities over time

Similarly, we compute the given user’s physical activities (weekly average of step counts) in all school weeks. Then, we compute the Spearman correlation between the user’s physical activities and time. We do so for all users and find those whose physical activities are significantly correlated with time (*p*<0.01). We denote this set of users as PA_T. Just as above, we study whether the number of users in PA_T is statistically significantly larger than the number of users who could be identified at random.

### Detecting co-evolution relationship between users’ network centralities and physical activities

For a given centrality measure and a given user in CoU, we compute the Spearman correlation between the user’s centrality values and physical activities (i.e., the step counts) over time. We do so for all users and find those whose centrality values are significantly correlated with physical activities (*p*<0.01). We denote the set of users for which at least one centrality measure significantly correlates with physical activities as NET_PA. Note that for a given user, we remove their centrality value in a given week if their physical activity has a missing value in that week. In other words, when computing correlations, the weeks with missing values are ignored.

Using the same approaches discussed above, we study: 1) the statistical significance of the number of users in NET_PA; 2) the number of users who are identified by exactly *k* (*k*=1,2,...10) centrality measures; 3) the number of users who are uniquely identified by a given centrality measure; and 4) the overlap between users identified by each pair of centrality measures.

### Examining trait differences between different sets of users

We study whether users in NET_T, PA_T, and NET_PA have different values of the seven static traits collected via surveys: agreeableness, conscientiousness, neuroticism, openness, extraversion, depression, and anxiety (“Individuals’ static trait data from surveys” section). This might reveal potential links between individuals’ static traits (e.g., personality), network positions, and physical activities.

We compare trait values for users in NET_T, PA_T, and NET_PA versus users who are not present in any of those sets (control group). For each of the three sets, for each of the seven static traits, we use Wilcoxon rank-sum test to examine whether trait values of users from the given set are significantly different from those of the control group. Here, we use the *p* value threshold of 0.05.

In addition to doing the above for each of NET_T, PA_T, and NET_PA user groups, we consider the following user groups as well:

1. Users who are in NET_T but not in PA_T (NET_T\PA_T).
2. Users who are in PA_T but not in NET_T (PA_T\NET_T).
3. Users who are in both NET_T and PA_T, (NET_T ∩PA_T).
4. Control group for user sets in items 1-3, consisting of those users who are absent from the union of these three sets, i.e., those users for which none of centralities or physical activities are significantly correlated with time.
5. Users who are in NET_T ∩PA_T but not in NET_PA ([NET_T ∩PA_T]\NET_PA).
6. Users who are in NET_PA but not in NET_T ∩PA_T (NET_PA\[NET_T ∩PA_T]).
7. Users who are in both NET_T ∩PA_T and NET_PA (NET_T ∩PA_T ∩NET_PA).
8. Control group for user sets in items 5-7, consisting of those users who are absent from the union of these three sets, i.e., those users for which centralities and physical activities are not correlated and do not both evolve with time.

## Results

### Dynamic network construction and basic network size and connectivity statistics

We construct a dynamic network consisting of the 576 users in NetU and their SMS communications during the first year period of the NetHealth study. The network consists of weekly snapshots. For each snapshot, two nodes are connected by an edge if they have exchanged at least one SMS in a given week (“Network construction” section).

To get a basic understanding of our constructed dynamic network in terms of its network size and connectivity, for each of its snapshots, we study the number of non-isolated nodes, the number of edges, and how many nodes are in the largest connected component (LCC).

**Fig. 2:**
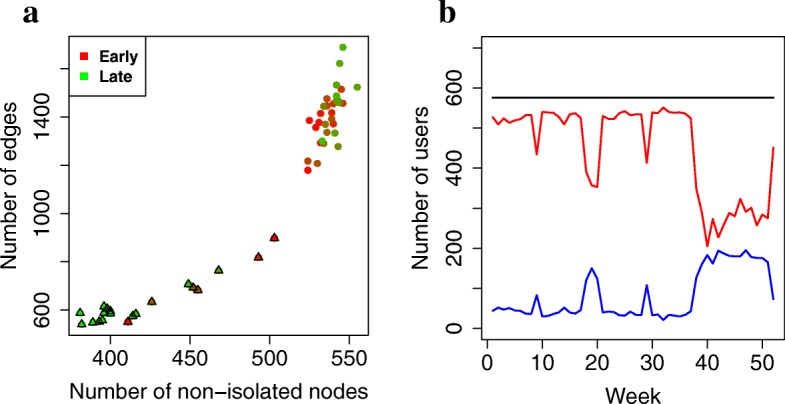
Network size statistics. **a** The number of non-isolated nodes and edges in each snapshot. Dots represent network snapshots of school weeks. Triangles represent network snapshots of break weeks. The color shifts from red to green as time goes from the first week (early) to the last week (late). **b** Some statistics about the connectivity of the dynamic network. The red line shows the number of nodes in the largest connected component (LCC) in each week. The blue line shows the number of isolated nodes in each week (i.e., users that have no links in the given week and are thus absent from that week’s network snapshot). The black line shows the maximum possible number of non-isolated nodes in each week, i.e., the total number of considered users (the size of NetU)

We find that network size varies between the different snapshots, with school week snapshots having more non-isolated nodes as well as edges than break week snapshots (Fig. 2). This is to be expected, since students tend to communicate with each other more often during school periods, when they are all on campus, than during break weeks, when they are off campus.

Also, we find that for school week snapshots, around 90% of all 576 nodes (NetU) are in the LCCs, which resembles many real-world networks (Kumar et al. 2010). In school week snapshots, most of the nodes that are not in the LCCs are isolates rather than forming small subgraphs that are disconnected from the LCCs.

For break week snapshots, we find that fewer nodes are in the LCCSs compared to school week snapshots, which is expected. Also, we find that the longer the break period, the fewer nodes are participating in the LCCs: around 75% of all users are in the LCCs during the 1-week long fall and spring breaks, around 60% of all users are in the LCCs during the several weeks long winter break, and only around 50% of all users are in the LCCs during the 3-month long summer break (Fig. 2).

### In-depth analysis of global network structure

Here, we analyze the global structure of our dynamic network in more detail. In particular, we study whether global topological properties of the network snapshots change with time, what the best fitting network model (i.e., random graph family) for each snapshot is, i.e., does the best-fitting model change with time, and whether some snapshots are more similar than others. Our key findings are summarized in Table 2 and detailed results are discussed in the following sections.

**Table 2:** Summary of our results

Global properties of network snapshots	Local properties of nodes	Trait differences between different user groups
∙ All snapshots have high node overlaps, but only snapshots from consecutive time periods have high edge overlaps	∙ Different centrality measures are complementary - they identify different users	∙ Users in NET_T do not show any trait difference compared to the control group
∙ Global network properties are stable over time	∙ This results in 222 NET_T users, 88 PA_T users, and 81 NET_PA users	∙ However, NET_T users who are also in PA_T are more likely to be introverted
∙ The best fitting network model for all snapshots is the same (GEO)	∙ The numbers of users in NET_T, PA_T, and NET_PA are statistically significantly high	∙ In addition, a subset of these (NET_T ∩PA_T) users who are also in NET_PA are also more likely to be anxious

#### Global network properties of snapshots are stable with time

**Fig. 3:**
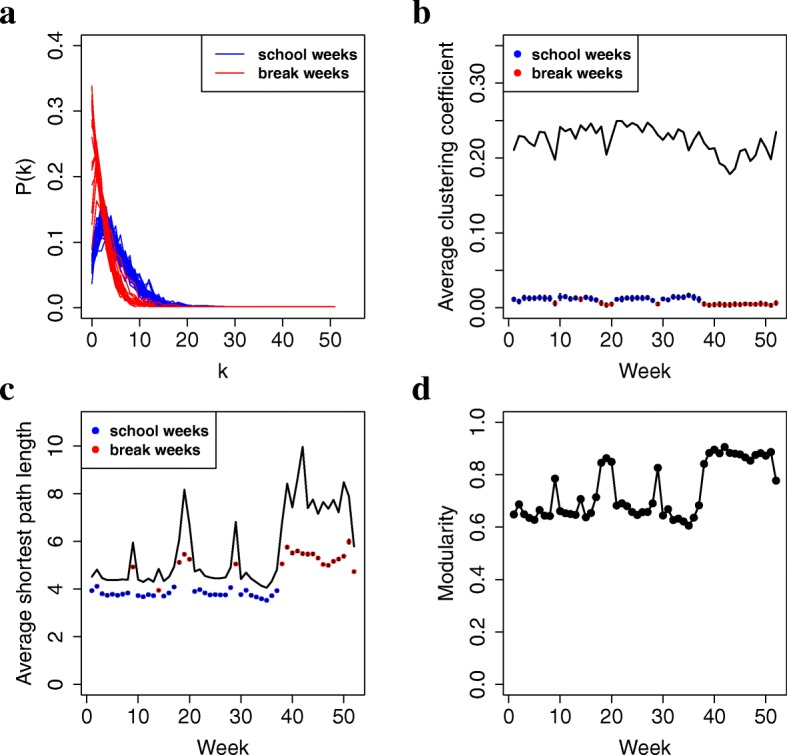
Global properties of the school or break week network snapshots: **a** degree distributions; **b** average clustering coefficients (solid line); **c** average shortest path lengths (solid line); and **d** modularity. In panels **b** and **c**, dots represent average clustering coefficients and average shortest path lengths, respectively, that would be expected by chance in networks of the same size, according to the degree distribution-preserving random graph model (ERDD)

We compute several popular global properties of network snapshots at different time points. We find that although global properties of school week snapshots are different than those of break week snapshots, the properties of the different school week snapshots are relatively similar, and so are the properties of the different break week snapshots (Fig. 3). More details are as follows.

First, school weeks and break weeks have different degree distributions (Fig. 3). But focusing either only on school weeks or only on break weeks, degree distributions of the different snapshots are similar. Existing work argues that many social networks follow scale-free degree distributions (Mislove et al. 2007). In our case, break weeks have power-law-like degree distributions, but school weeks’ degree distributions deviate from the power-law to some extent.

Second, school weeks and break weeks have different average clustering coefficients, average shortest path lengths, and modularities (Fig. 3). But focusing either only on school weeks or only on break weeks, these properties do not significantly change with time.

Third, our network snapshots have the small-world phenomenon characterized by a high average clustering coefficient (of around 0.2 for our data) and small average shortest path length (between around four and eight for our data). The small-world phenomenon has been observed in many other social networks (Bonato et al. 2010; Watts and Strogatz 1998; Watts 1999. It is interesting that both the average clustering coefficients and the average shortest path lengths of our network snapshots are statistically significantly higher than expected by chance in the networks of the same sizes (*p*-value <0.01) (Fig. 3), while typically, the higher the average clustering coefficient, the lower the average shortest path length, and vice versa. Our result suggests that our network snapshots might have a number of smaller highly densely connected regions (resulting in high average clustering coefficients) that are separated from each other via few interactions, i.e., via sparse network regions (resulting in higher than random average shortest path length). This is confirmed by high modularity (between around 0.6-0.9 in our data, Fig. 3).

It might be surprising that modularity is higher during break weeks than during school weeks, given that break week snapshots have fewer nodes and edges than school week snapshots, and thus, one might expect fewer (near-)cliques during breaks when students are off-campus. However, this might not be surprising. First, modularity accounts for network size. Second, even though more nodes are isolated in break weeks than in school weeks, those nodes that do interact in break weeks might correspond to closer (and thus more densely interconnected) friend groups than nodes that interact in school weeks. In other words, SMS interactions during breaks might be more indicative of true (strong) friendships than SMS interactions during school weeks.

For other global properties, such as the clustering spectra and shortest path length distributions of the network snapshots, see Additional file 1: Figure S4.

#### Network snapshots at different time points belong to the same graph family

**Fig. 4:**
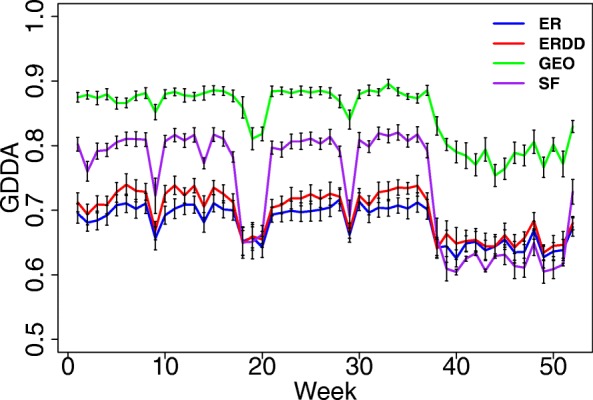
The fit of four network models (ER, ERDD, GEO, and SF) to each network snapshot with respect to GDDA. For each model, each data network snapshot is compared to 30 random network instances, and the fit is averaged over the 30 instances. Error bars correspond to the standard deviations. The higher the value of GDDA, the better the fit

Here, we compare the fit of the network snapshots to four network models (ER, ERDD, GEO, and SF) with respect to GDDA (“Evaluating the fit of the dynamic network to different network models, i.e., graph families” section). Our goal is to evaluate whether the best fitting model changes with time. If not, that would further indicate stability of global network structure with time. Indeed, we again confirm the stability, because we find that the best-fitting model does not change with time (Fig. 4). Specifically, the best fitting model is GEO (and not SF, as one might expect). This observation agrees with the existing literature that argues that geometric random graphs (GEO) satisfy many properties of real-world social networks (Bonato et al. 2010; Bradonjić et al. 2008; Bonato et al. 2015).

#### Similarities between network snapshots at different time points

In this section, we compute similarities between all pairs of network snapshots with respect to 15 measures (NO, EO, GCD, GDDA, RGFD, and the 10 centrality measures, “Computing similarities between network snapshots at different time points” section) in order to study whether network snapshots of school weeks and break weeks are similar and whether consecutive snapshots are more similar than non-consecutive snapshots. 15 measures can be classified into three groups: 1) network size-related measures, NO and EO, 2) graphlet-based measures, GCD, GDDA, and RGFD, and 3) the 10 centrality-based measures.

**Fig. 5:**
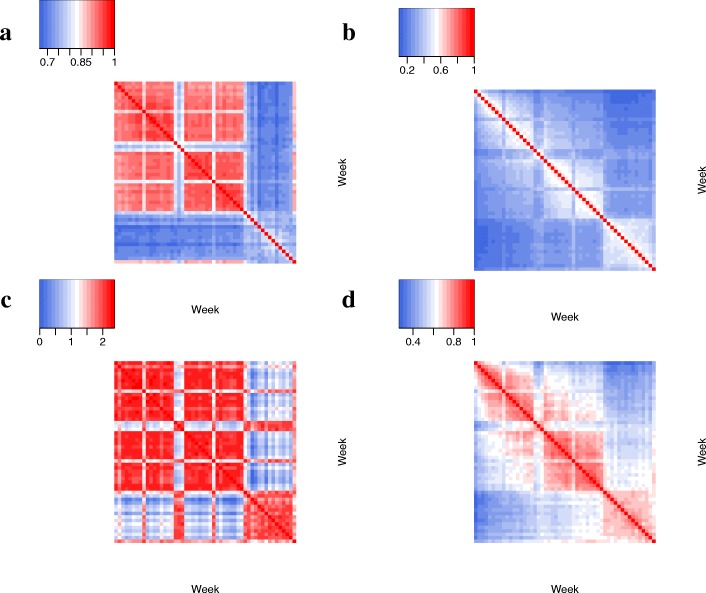
Pairwise network similarities with respect to four measures: **a** node overlap (NO); **b** edge overlap (EO); **c** graphlet correlation distance (GCD); **d** correlation between degrees (DEGC) over all nodes. Other global (GDDA and RGFD) and local network properties (i.e., the remaining centrality measures) show similar trends. Each row/column of a similarity matrix corresponds to the network snapshot of a given week. Values on the diagonals are always 1, because each network snapshot is identical to itself. In each panel, the higher the matrix value, the higher the snapshot similarity. Please note that the different panels do not necessarily use the same color scheme. In each panel, the week number increase from left to right, and from top to bottom

First, we find that school week snapshots are more similar to each other than they are to break week snapshots, with respect to all 15 network similarity measures (Fig. 5). Second, we find that in terms of EO and centrality-based measures, snapshots that are consecutive in time are more similar to each other than non-consecutive snapshots, while for NO and the graphlet-based measures, this is not necessarily the case (Fig. 5). This indicates that different measures have difference in measuring network similarities.

**Fig. 6:**
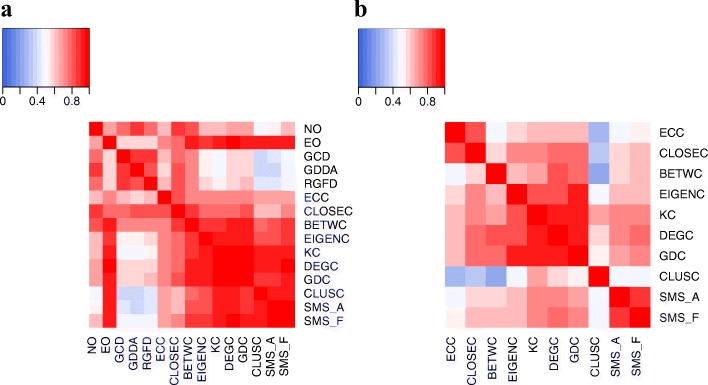
Correlations between different global and local measures. **a** Spearman correlations between all pairs of the considered network similarity measures obtained by comparing the measures’ snapshot similarity matrices. **b** Spearman correlations between all pairs of the considered centrality measures obtained by comparing the centrality values over all nodes in all network snapshots. In each panel, the higher the matrix value, the higher the similarity between the corresponding measures. Please note that the different panels do not necessarily use the same color scheme

To study relationships between each pair of the 15 considered measures, we compute Spearman correlations between their snapshot similarity matrices (“Computing similarities between network snapshots at different time points” section). We find that the 15 measures are all significantly correlated (*p*<0.05). However, correlation values vary between the different measures. Among the different graphlet-based measures, the pairwise correlations are high (average *r*=0.86). This is not surprising given that they are all based on graphlets and differ mostly in how they mathematically compare two network snapshots based on their graphlet patterns. Similarly, among the different centrality-based measures, the pairwise correlations are also high (average *r*=0.85). But the pairwise correlations between the graphlet-based measures and the centrality-based measures are lower (average *r*=0.5) (Fig. 6). This indicates that graphlet-based and centrality-based measures do have difference in measuring network similarities. In terms of the network size-related measures (NO and EO), their pairwise correlation is not high (*r*=0.65). Among NO and EO, NO is more similar to the graphlet-based measures (average *r*=0.8) than to the centrality-based measures (average *r*=0.66). On the contrary, EO is more similar to the centrality-based measures (*r*=0.89) than to the graphlet-based measures (average *r*=0.56) (Fig. 6).

Since we observe high correlations between the snapshot similarity matrices of the different centrality-based measures, we wonder whether the different centrality measures are potential redundant to each other. Here, to study potential redundancies of the different centrality measures, we compute, for each pair of centrality measures, the Spearman correlation between their centrality values over all nodes in a network snapshot, and by averaging Spearman correlations over all snapshots. As the outcome, we observe high correlations between some but not all measures (Fig. 6). For example, DEGC, KC, and GDC are highly correlated (average *r*=0.88). This is not surprising as they are all based on related notions of degree or graphlet degree of a node. Also, CLOSEC and ECC are highly correlated (*r*=0.84), likely because both measures are based on the concept of shortest paths. Similarly, SMS activity and SMS frequency are highly correlated (*r*=0.87), which again is not surprising because the two measures capture the same information in slightly different mathematical ways. Surprisingly, BETWC, which is based on shortest paths, is more similar to the degree-based measures (DEGC, KC, and GDC) than to the other shortest path-based measures (CLOSEC and ECC), which agrees with a recent study in the computational biology domain (Faisal and Milenković 2014). The only remaining centrality measure, CLUSC, does not correlate well with any other centrality measure, except perhaps with KC and somewhat with DEGC.

In summary, although we observe high correlations between centrality measures that are based on similar topological “principles”, 23 out of all 45 pairs of centralities have correlations below 0.65. This indicates that the considered centrality measures are at least somewhat complementary to each other, even though they are highly correlated according to the above analysis.

### Identification of users whose network centralities evolve over time

In this section, we aim to answer whether social network positions of the nodes evolve over time. For each of the 10 considered network position (centrality) measures (“Local network centralities” section), for each node, we compute a given node’s centrality value in each snapshot. (For an illustration of the nodes’ degrees in each snapshot, see Additional file 1: Figure S5.) Then, we compute Spearman correlation between the given user’s centrality values and time (“Finding users whose network centralities evolve over time” section).

We find that for at least one centrality measure, centrality values are statistically significantly correlated with time (*p*<0.01) for 222 (74%) out of the 302 users in CoU. We denote this set of users as NET_T. Importantly, the number of NET_T users is statistically significantly larger (*z*-score =21) than the number of users that could be identified from randomized data (29 users, Additional file 1: Figure S6).

To understand whether in the above analysis the different centrality measures identify different users, we study 1) whether any users in NET_T are found by more than one or even all of the centralities, 2) how many users are uniquely identified by each centrality, and 3) the pairwise overlaps of users identified by the different centralities. We expect that the different centralities could identify at least somewhat different sets of users, but that at least some users would be supported by multiple centralities.

**Fig. 7:**
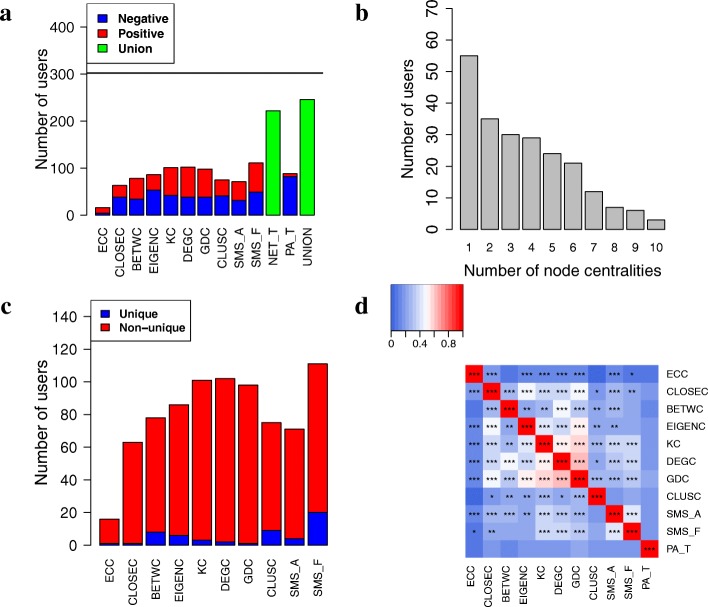
Results regarding users whose network centralities or physical activities change with time. **a** The number of significantly-changing users for each of the 10 centralities individually (ECC to SMS_F) or by at least one of them (NET_T), for physical activity (PA_T), and for the union of NET_T and PA_T (the last bar). Red and blue portions of the given bar indicate users that are positively and negatively correlated with time, respectively. **b** The number of users found by exactly *k* centralities (*k* = 1, 2,..., 10). **c** The number of users that are unique to the given centrality, i.e., for a given user, only for this centrality, the user’s centrality values significantly change with time. **d** Pairwise overlaps between significantly-changing users predicted by the different centralities or physical activity. An overlap is measured as Jaccard index, and the resulting overlap size is indicated by the given color. The significance of the overlaps is computed by using the hypergeometric test. *, **, and *** indicate statistical significance at *p*-value thresholds of 0.05, 0.01, and 0.001, respectively

Indeed, we find that 167 (75%) of the 222 NET_T users are supported by multiple centralities, while the remaining users are supported by a single centrality (Fig. 7). Moreover, each centrality identifies a small number of significantly-changing users that are unique to the given centrality (Fig. 7). Furthermore, when we study pairwise overlaps of significantly-changing users found by the different centralities, we observe that most of them are significantly overlapping (*p*<0.01), but very few of them have high absolute overlaps, e.g., Jaccard index of above 0.8 (Fig. 7). The statistically significant overlaps are encouraging, as they increase the credibility of users in NET_T supported by multiple centrality measures. Yet, given the low absolute overlap sizes, the different centralities are not redundant to each other. In other words, the different centrality measures seem to capture at least somewhat complementary user sets, which is why for our subsequent analyses, we focus on their union, i.e., NET_T.

### Identification of users whose physical activities evolve over time

In this section, we aim to answer whether physical activities of the NetHealth participants evolve over time. For each user in CoU, we compute the Spearman correlation between the given user’s physical activities and time (“Examining the change of users’ physical activities over time” section). We find that physical activities are statistically significantly correlated with time (*p*<0.01) for 88 (29%) out of the 302 users in CoU (Fig. 7). We denote this set of users as PA_T. The number of users in PA_T is statistically significantly higher (*z*-score =44) than the number of users that we can identify from randomized data (2 users, Additional file 1: Figure S6). PA_T does not significantly overlap with centrality-changing users identified by the different centrality measures (Fig. 7).

### Identification of users whose network centralities and physical activities co-evolve over time

In the previous sections, we have identified users whose network positions or physical activities significantly correlate with time. Since time only increases, significant (positive or negative) correlation with time implies that the identified users’ network positions or physical activities increase or decrease over time. In this section, we study whether a co-evolution relationship exists between the given user’s network positions and physical activities. Such analysis not only considers cases where network positions and physical activities both increase or decrease over time, but also takes into account cases where network positions and physical activities co-evolve in alternative, e.g., wave-like patterns over time. Here, for a given user, a strong co-evolution relationship would mean that when the user’s network centrality values increase, the user’s physical activities also increase (positive correlation) or decrease (negative correlation).

**Fig. 8:**
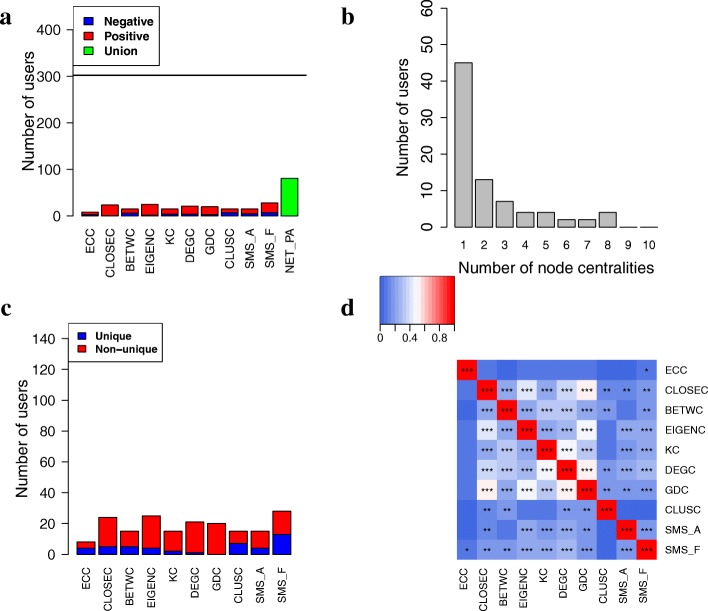
Results regarding users whose network centralities co-evolve with physical activities. **a** The number of users with co-evolution relationship for each of the 10 centralities individually (ECC to SMS_F) or by at least one of them (NET_PA). Red and blue portions of the given bar indicate users whose centralities are positively and negatively correlated with physical activities, respectively. **b** The number of users found by exactly k centralities (*k*=1,2,…,10). **c** The number of users that are unique to the given centrality, i.e., for a given user, only for this centrality, the user’s centrality values are significantly correlated with physical activities. **d** Pairwise overlaps between users with co-evolution relationship predicted by the different centralities. An overlap is measured as Jaccard index, and the resulting overlap size is indicated by the given color. The significance of the overlaps is computed by using the hypergeometric test. *, **, and *** indicate statistical significance at p-value thresholds of 0.05, 0.01, and 0.001, respectively

For each centrality measure, for each user in CoU, we compute the Spearman correlation between the given user’s centrality values and physical activities (“Detecting co-evolution relationship between users’ network centralities and physical activities” section). We find that for at least one centrality measure, centrality values are statistically significantly correlated with physical activities (*p*<0.01) for 81 (27%) out of the 302 users in CoU (Fig. 8). We denote this set of users as NET_PA. Importantly, the number of NET_PA users is statistically significantly larger (*z*-score =10) than the number of users that could be identified from randomized data (28 users, Additional file 1: Figure S6).

To understand whether different centralities identify different users with co-evolution relationships, we study 1) whether any users in NET_PA are found by more than one or even all of the centralities, 2) how many users are uniquely identified by each centrality, and 3) the pairwise overlaps of users identified by the different centralities. Same as above, we expect that the different centralities could identify different sets of users, but that some users would be supported by multiple centralities. Indeed, we find that 45 (56%) of the 81 NET_PA users are supported by multiple centralities, while the remaining users are supported by a single centrality (Fig. 8). Moreover, each centrality uniquely identifies a number of users with co-evolution relationship (Fig. 8). Furthermore, when we study pairwise overlaps of users with co-evolution relationship found by the different centralities, we observe that most of them are significantly overlapping (*p*<0.01), but very few of them have high Jaccard index, e.g., of 0.8 or higher (Fig. 8). This means that although users identified by the different centrality measures are statistically significantly overlapping, the different centralities are not redundant to each other.

### Trait differences between the different user groups

Via the above analyses, we find three sets of users 1) NET_T: users whose centrality values are significantly correlated with time with respect to at least one centrality measure, 2) PA_T: users whose physical activities are significantly correlated with time, and 3) NET_PA: users whose centrality values are significantly correlated with physical activities over time, with respect to at least one centrality measure. In this section, we aim to identify user traits that are associated with temporal changes in network position and physical activity. We do this by contrasting static traits (the five personality traits, anxiety, and depression) of different user groups (see below) to the corresponding control groups (“Examining trait differences between different sets of users” section) and by analyzing potential trait differences between the different user groups.

#### Traits of users in NET_T, PA_T, and their subsets

**Fig. 9:**
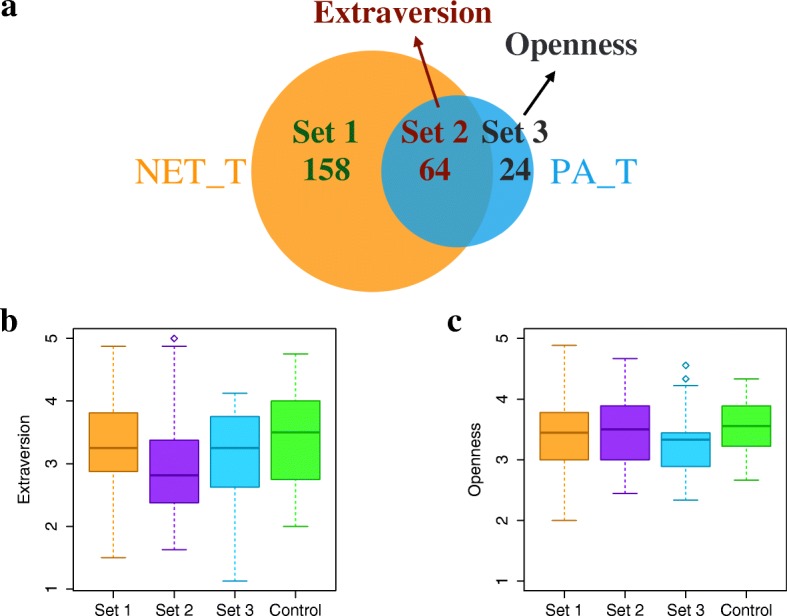
Trait differences between NET_T, PA_T and their subsets. **a** The overlap of NET_T and PA_T. Set 1 denotes users who are in NET_T but not in PA_T. Set 2 denotes users who are in both NET_T and PA_T. Set 3 denotes users who are in PA_T but not in NET_T. **b** Boxplots of extraversion scores of user sets 1-3 and a control set, where the latter denotes users from CoU who are not in any of sets 1-3. **c** Boxplots of openness scores of the same user sets as in panel **b**

In this part, we analyze five sets of users shown in Fig. 9: NET_T, PA_T, Set 1 (users in NET_T but not in PA_T), Set 2 (users in both NET_T and PA_T), and Set 3 (users in PA_T but not in NET_T). Our findings are as follows (Table 2).

First, although NET_T users do not show any trait difference compared to the control set, Set 2 users, i.e., a subset of NET_T who are also in PA_T, have significantly lower (*p*<0.05) extraversion scores than the control set (Fig. 9). Users with low extraversion scores tend to be retiring, reserved, and cautious; they are more likely to value tradition rather than stimulation (Roccas et al. 2002). Second, PA_T users already have significantly lower extraversion scores than the control set. Hence, it is not surprising that Set 2, a subset of PA_T that is in NET_T which contains a majority of PA_T users, also has lower extraversion scores compared to the control set. But Set 3, also a subset of PA_T, does not have significantly lower extraversion scores compared to the control set (Fig. 9). Instead, Set 3 users have significantly lower openness scores than the control set (Fig. 9). Users with low openness scores tend to be down-to-earth, insensitive, and conventional (Roccas et al. 2002).

In summary, by analyzing users who are in both NET_T and PA_T (i.e., Set 2, or NET_T ∩ PA_T),we obtain additional insights (i.e., trait differences) compared to analyzing the entire NET_T and PA_T sets or those users that are in exactly one of these two sets.

#### Traits of users in NET_T ∩PA_T, NET_PA, and their subsets

**Fig. 10:**
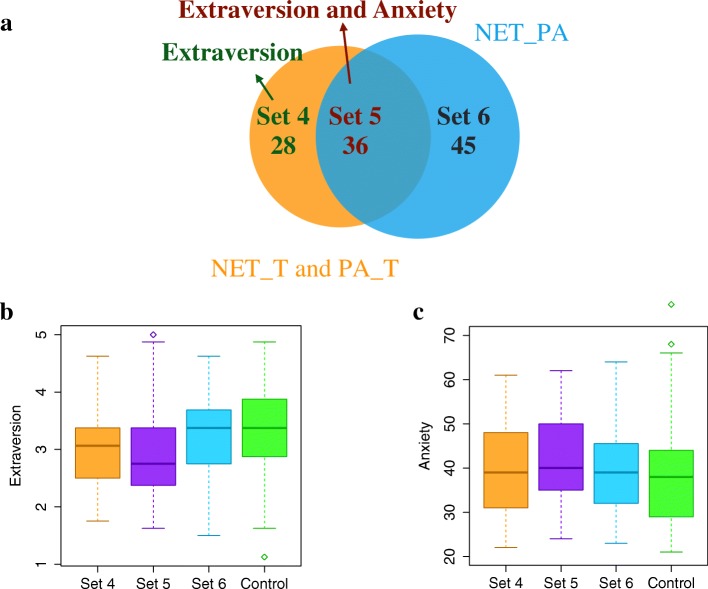
Trait differences between NET_T ∩ PA_T (i.e., set 2 from Fig. 9), NET_PA and their subsets. **a** The overlap of NET_T ∩ PA_T and NET_PA. Set 4 denotes users who are in NET_T ∩ PA_T but not in NET_PA. Set 5 denotes users who are in both NET_T ∩ PA_T and NET_PA. Set 6 denotes users who are in NET_PA but not in NET_T ∩ PA_T **b** Boxplots of extraversion scores of user sets 4-6 and a control set, where the latter denotes users from CoU who are not in any of sets 4-6. **c** Boxplots of anxiety scores of the same user sets as in panel **b**

In this part, we analyze five other sets of users shown in Fig. 10: NET_T ∩PA_T (users in the intersection of NET_T and PA_T, i.e., Set 2 from the previous analysis), NET_PA, Set 4 (users in NET_T ∩PA_T but not in NET_PA), Set 5 (users in both NET_T ∩PA_T and NET_PA, the “core”), and Set 6 (users in NET_PA but not in NET_T ∩PA_T). Note that for a given user, even if both the user’s centrality values and their physical activities are correlated with time (and hence the user is in NET_T ∩PA_T), this does not necessarily imply that the user’s centrality values and physical activities co-evolve over time (i.e., that the user is in NET_PA). For example, if a user’s centrality values are stable at first and then increase as time progresses, and the user’s physical activities increase at first and then become stable as time progresses, then the user would be in NET_T ∩PA_T but not in NET_PA, because the user’s centrality values and their physical activities do not change at the same time.

In terms of trait differences, first, we find that both Set 4 and Set 5 have significantly lower extraversion scores than the control set (Fig. 10). This makes sense because NET_T ∩PA_T, the union of Set 4 and Set 5, has significantly lower extraversion scores, per our above analysis. Interestingly, in addition to extraversion, Set 5 users also have significantly higher anxiety scores (Fig. 10) than the control group, which we do not observe in Set 4 or in NET_T ∩PA_T. Second, NET_PA does not show any trait differences compared to the control set, but Set 5, a subset of NET_PA, has lower extraversion scores and higher anxiety scores. Note that anxiety scores range from 20 to 80, where the higher the score, the greater the anxiety (Grös et al. 2007).

In other words, by analyzing users who are in both NET_T ∩PA_T and NET_PA (i.e., Set 5),we obtain additional insights (i.e., trait differences) compared to analyzing the entire NET_T ∩PA_T and NET_PA sets or those users that are in exactly one of these two sets.

Here, we summarize sets of users that show significantly difference traits scores compared to the control (Table 2):

- Users whose both centrality values and physical activities change with time (in both NET_T and PA_T) have significantly lower extraversion scores (i.e., are more likely to be introverted).
- Users whose physical activities but not centrality values change with time (in PA_T but not in NET_T) have significantly lower openness scores (i.e., are less likely to be open).
- Users whose both centrality values and physical activities change with time and the two also co-evolve together (in all of NET_T, PA_T, and NET_PA) have significantly lower extraversion scores and higher anxiety scores (i.e., are more likely to be introverted and anxious).
- Users whose both centrality values and physical activities change with time but do not co-evolve together (in NET_T and PA_T, but not in NET_PA) have significantly lower extraversion scores (i.e., are more likely to be introverted).

## Conclusions

In this work, we first select the time period and user pool of interest. Then, we construct a high-quality dynamic network of social (SMS) interactions between compliant users. The dynamic network is represented by weekly network snapshots in which two users are connected if they exchange at least one SMS in the given week. We study evolution of the dynamic network by comparing global properties of network snapshots and local properties (centralities) of nodes at different time points. In terms of global properties, we find that: 1) several commonly used global properties are stable over time (except for break weeks), 2) the best fitting model for each snapshot is the same, which is GEO, and 3) consecutive snapshots are more similar than non-consecutive snapshots with respect to EO and centrality-based measures. Although global properties of network snapshots do not change with time, local properties of nodes do change with time. We find that for at least one centrality measure, centrality values are statistically significantly correlated with time for 222 (74%) out of the 302 users in CoU (users that are compliant in terms of both SMS and Fitbit data), and it is unlikely to get as high number of users by chance. The 222 users form NET_T. Analogously, we find that physical activities are statistically significantly correlated with time for 88 (29%) out of the 302 users in CoU, and it is unlikely to get as high number of users by chance. The 88 users form PA_T. Furthermore, we study the co-evolution relationship between the users’ centrality values and physical activites. We find that for at least one centrality measure, centrality values are statistically significantly correlated over time with physical activities for 81 (27%) out of the 302 users in CoU, and it is unlikely to get as high number of users by chance. The 81 users form NET_PA. Last, we examine trait differences between users in NET_T, PA_T, NET_PA, and their intersections. We find that: 1) users whose both centrality values and physical activities change with time are more likely to be introverts, 2) users whose physical activities but not centralities change with have significantly lower openness scores, 3) users whose both centrality values and physical activities change with time and co-evolve together are more likely to be introverts and anxious individuals, and 4) users whose both centrality values and physical activities change with time but do not co-evolve together are more likely to be introverts.

Our work could be extended in many aspects in the future. For example, currently, we study the first-year period of the NetHealth study. Our network analysis framework could be applied to its subsequent periods (year two or three), or to datasets from other, non-student populations. Also, while in this paper we study evolving physical activities as representative health-related behaviors, one could examine other Fitbit-based behaviors, such as sleep habits or heart rates. Moreover, in this study, after identifying individuals with specific network or physical activity temporal profiles, we have begun the process of understanding who is more likely to have specific profiles. We have identified some personal traits (extroversion, openness, anxiety) as being associated with specific temporal profiles. Whether and how these and other personal traits are associated with specific temporal profiles is an important avenue for future research. Models relating personal traits to temporal network and activity profiles will need to take into account that personal traits can change in response of changes in network position and activity levels (and vice versa) (Schwaba and Bleidorn 2018; Cobb-Clark and Schurer 2012). As such, this paper provides the foundation for developing predictive models that would use longitudinal network and health data to guide individuals’ well-being. For example, one could build machine learning models that would predict individuals’ health-related behaviors from their networks at a given time point and vice versa, or individuals’ future networks or behaviors from their past networks or behaviors.

## Additional files

Additional file 1 Supplementary material. (PDF 475 kb)

Additional file 2 Data. (ZIP 375 kb)

## Abbreviations

BETWC: Betweenness centrality
CESD: Center for epidemiological studies depression scale
CLUSC: Clustering coefficient
CLOSEC: Closeness centrality
CoU: Users that are compliant in terms of both SMS and Fitbit data
DEGC: Degree centrality
ECC: Eccentricity centrality
EIGENC: Eigenvector centrality
EO: Edge overlapping
ER: Erdős–Rényi random graphs
ERDD: Generalized Erdős–Rényi random graph model with preserved degree distribution
GCD: Graphlet correlation distance
GDC: Graphlet degree centrality
GDD: Graphlet degree distribution
GDDA: Graphlet degree distribution agreement
GEO: Geometric random graphs
KC: K-coreness centrality
LCC: Largest connected component
NET_PA: Users whose at least one centrality values are significantly correlated with physical activities
NET_T: Users whose at least one centrality values are significantly correlated with time
NetU: Users that are compliant in terms of SMS data
NO: Node overlapping
PA_T: Users whose physical activities are significantly correlated with time
RGFD: Relative graphlet frequency distance
SF: Scale-free networks
SMS_A: SMS activity
SMS_F: SMS frequency
STAI: State-Trait Anxiety Inventory
