# Supplementary material for: Network analysis of the NetHealth data: exploring co-evolution of individuals’ social network positions and physical activities
Source: Appl Netw Sci. 2018 Nov 2;3(1):45. doi: 10.1007/s41109-018-0103-2 (PMC6223883; doi:10.1007/s41109-018-0103-2)
Supplement: Supplementary file 1 — Supplementary material. (PDF 475 kb) [file 41109_2018_103_MOESM1_ESM.pdf]

RESEARCH

Supplementary Material for: Network analysis of the NetHealth data: Exploring co-evolution of individuals' social networks and physical activities

Shikang Liu[1], David Hachen[2], Omar Lizardo[2], Christian Poellabauer[1], Aaron Striegel[1] and Tijana Milenković[1,3,4*]

*Correspondence: tmilenko@nd.edu
[1]Department of Computer Science and Engineering, University of Notre Dame, Notre Dame, IN 46556, USA
Full list of author information is available at the end of the article

S1

We filter out users who formally dropped out of the study or became completely inactive (i.e., did not send or receive any SMSs) within the first year period. This leaves us with 576 study participants as our user pool for constructing the network. We denote this pool of users as NetU. Here, we evaluate SMS activities of users in NetU by checking the number of weeks in which a given user is active (i.e., send or receive at least one SMS in a given week). We find that most users in NetU are active in most of the weeks during the first year period (Figure S2). This means that instead of sending/receiving SMSs occasionally for a short period then disappear, most NetU users are constantly active throughout the study period, especially during school weeks. Thus, our user pool is of good quality in terms of long-term SMS activities.

S2

We vary values of the two parameters, time interval Δt and link threshold w, in order to empirically choose for constructing the network. We expect our network to be sparse (i.e., low density) and has large LCCs, which resembles many real-world networks. By examining different combinations of Δt and w, we choose Δt = 1 week and $w = 1$. With this choice, LCC sizes of school weeks are high (above 0.9), and LCC sizes of break weeks are significantly lower than school weeks (Figure S3). In addition, The densities of snapshots are low, and densities of break weeks are significantly lower than school weeks (Figure S3). Thus, network snapshots we constructed resemble real-world networks in terms of density and LCC size. Moreover, they capture valuable temporal information (e.g., fluctuations in break weeks) from the data.

S3

As explained in Section Studying the evolution of global properties of network snapshots in the main paper, we analyze commonly used global network properties of each network snapshot. Definitions of these properties are as follows.

- Degree distribution is the probability distribution of degrees of nodes over the whole network

- Average clustering coefficient is the average of the clustering coefficients of all the nodes in the network. The clustering coefficient of a node is the proportion of edges between the nodes within its neighbourhood divided by the number of edges that could possibly exist between them ;
- Clustering spectrum is the distribution of average clustering coefficient for nodes with each degree k;
- Average shortest path length is the average of shortest path lengths for all possible pairs of nodes in the network;
- Shortest path length distribution is the distribution of shortest path lengths for all possible pairs of nodes in the network.
- Modularity is the fraction of the edges that fall within the given groups minus the expected fraction if edges were distributed at random [1].

S4

As explained in Section Local network centralities in the main paper, we measure social network positions of all nodes in each snapshot via 10 centrality measures, each of which aims to capture the importance of a node in a network, often from a complementary perspective compared to others. Definitions of traditional network centrality measures are as follows.

- Degree centrality (DEGC) measures the degree of a node in the network,i.e. the number of the node's neighbors. The higher the degree of a node, the more central the node according to DEGC. Most real-world networks have 'power-law' degree distributions, with many low degree nodes and few high-degree nodes (hubs) [2].
- K-core of a network is a maximal subset of nodes in the network such that each node is connected to at least k others in the subset. K-coreness centrality (KC) of a node is k if the node is in k-core.
- Graphlet degree centrality (GDC) measures how many graphlets anode participates in, for all 2–5-node graphlets [3]. Intuitively, the more graphlets a node touches, the more central the node is according to GDC. Because it captures the extended network neighborhood of a node, GDC is a highly sensitive measure of network topology.
- Eigenvector centrality (EIGENC) is an extension of degree centrality. A node with high eigenvector centrality score means that a node is connected to many nodes who themselves have high cores.
- Betweenness centrality (BETWC) measures the involvement of a node in the shortest paths in the network. Intuitively, nodes that occur in many shortest paths have high centrality according to BETWC.
- Closeness centrality (CLOSEC) measures the 'closeness' of a node toall other nodes in the network. Intuitively, nodes with small shortest pathdistances to all other nodes have high centrality according to CLOSEC.
- Eccentricity centrality (ECC) is related to CLOSEC, except that it measures the 'closeness' of a node only to the farthest node in the network [4]. Intuitively, nodes with small shortest path distances to the furthest node in the network have high centrality according to ECC.

- Clustering coefficient centrality (CLUSC) measures, for a given node, how many pairs of neighbors of the node are connected by an edge, out of all pairs of the node's neighbors. Intuitively, the more interconnected the neighborhood of the node, the more central the node is according to CLUSC.

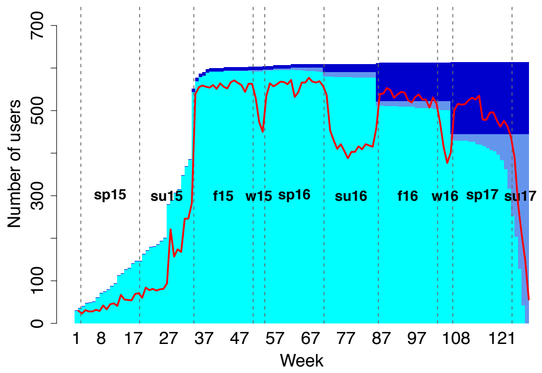

Figure S1: **The summary of users' SMS activities** Blue (all shades combined): Active (as sender or receiver) at least once prior to week i (inclusive); Dark blue: Dropped prior to week i (inclusive) (out of non-dropped, as of the end of the semester of week i); Mid-shade blue: Active last time prior to week i (inclusive) (out of non-dropped, as of the end of the semester of week i); Red line: Active in week i (out of all 615 users)

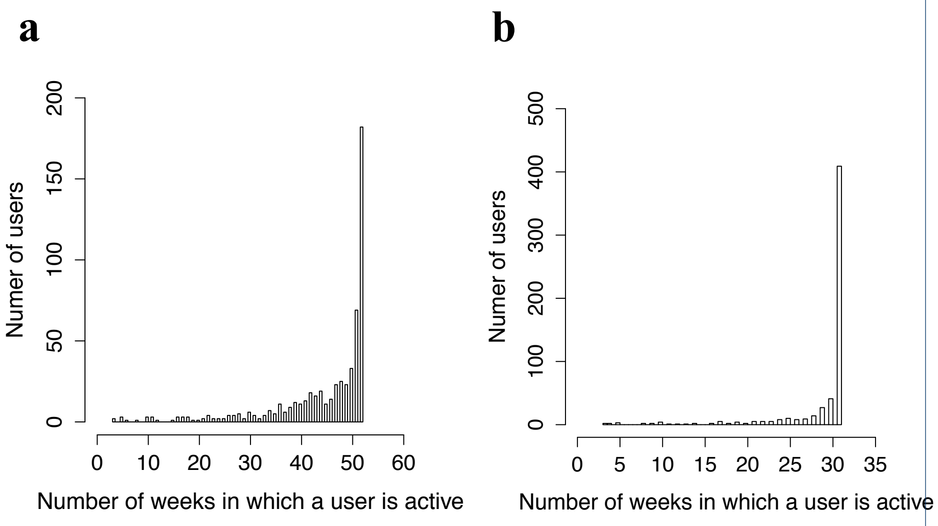

Figure S2: **Evaluation of SMS activities for users in NetU. (a)** Number of weeks in which a user is active during the first year period. By active, we mean send or receive at least one SMS in a given week. **(b)** Number of weeks in which a user is active during school weeks in the first year period.

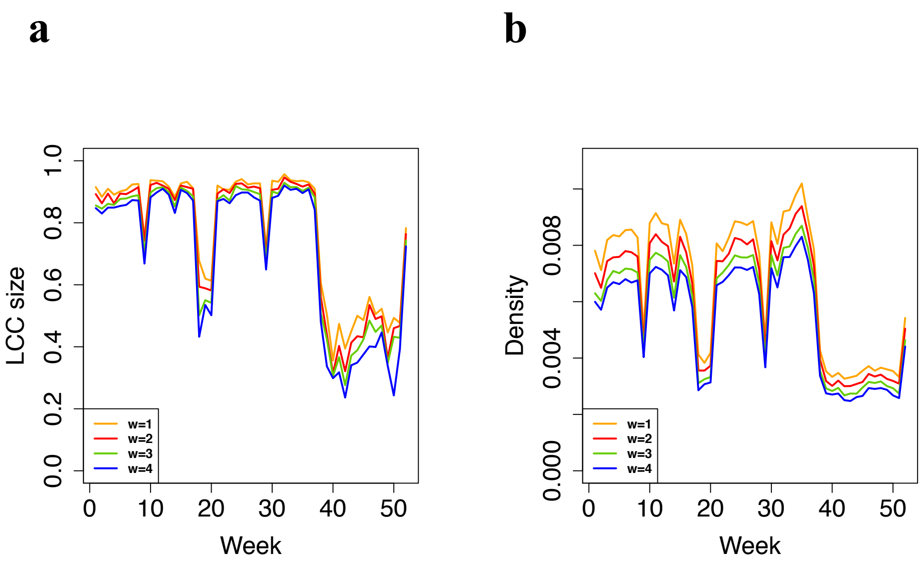

Figure S3: **LCC size and density for each snapshot.** The time interval for each snapshot is one week. w is the weight threshold, i.e., two nodes are connected if they have at least w SMS events in a given week. **(a)** The LCC size for each snapshot. **(b)** The density for each snapshot.

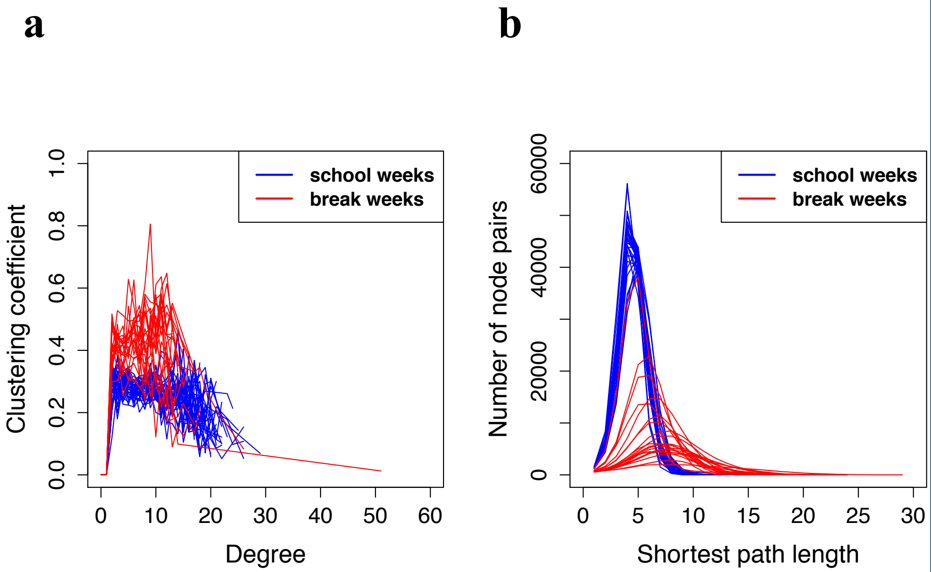

Figure S4: **Global properties of network snapshots. (a)** The clustering spectra. y axis is the average clustering coefficient for nodes with a given degree k. **(b)** The distribution of shortest path lengths. In (a) and (b), blue lines denote for school weeks, and red lines denote for break weeks.

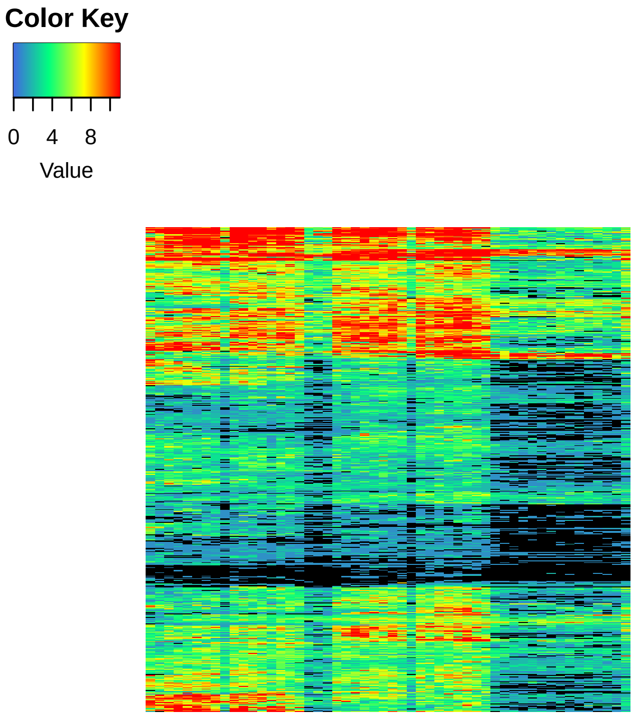

Figure S5: **Illustration of degrees of nodes in each snapshot.** Columns represents for weeks (snapshots) during the first year period. Rows represents for users that are compliant in terms of SMS data. Values represent for degrees of nodes in each snapshot. We observe clusters of nodes that particular evolving patterns (e.g., degree increasing or decreasing with time).

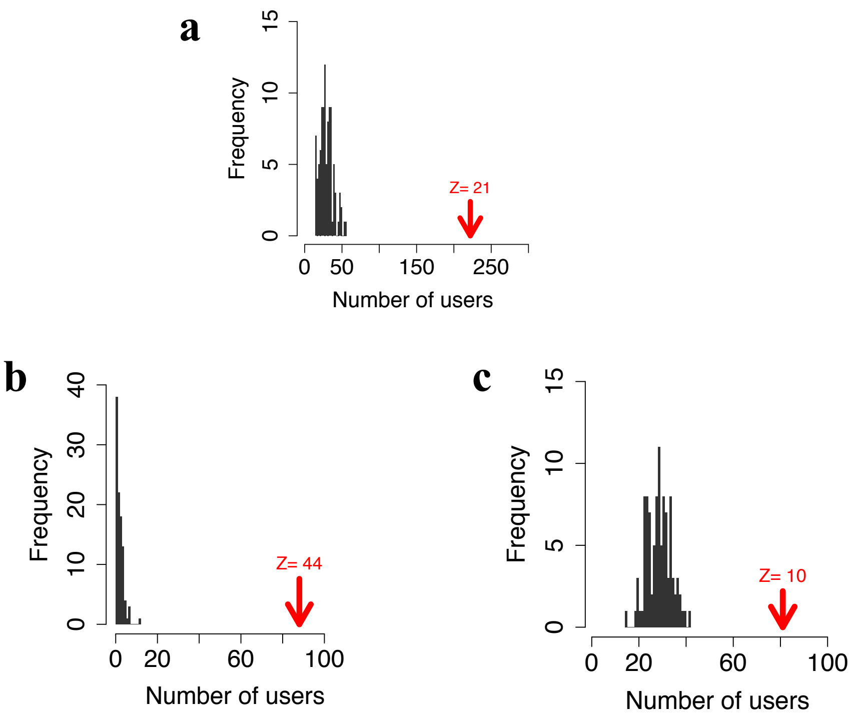

Figure S6: **Statistical significance of NET_T, PA_T and NET_PA. (a)** The number of users in NET_T compared to random instances. **(b)**The number of users in PA_T compared to random instances. **(c)** The number of users in NET_PA compared to random instances.

Author details
[1]Department of Computer Science and Engineering, University of Notre Dame, Notre Dame, IN 46556, USA. [2]Department of Sociology, University of Notre Dame, Notre Dame, IN 46556, USA. [3]Eck Institute for Global Health, University of Notre Dame, Notre Dame, IN 46556, USA. [4]Interdisciplinary Center for Network Science and Applications (iCeNSA), University of Notre Dame, Notre Dame, IN 46556, USA.

References

1. Newman, M.E.: Modularity and community structure in networks. Proceedings of the national academy of sciences **103**(23), 8577–8582 (2006)
2. Albert, R., Barabási, A.-L.: Statistical mechanics of complex networks. Reviews of Modern Physics **74**(1), 47 (2002)
3. Milenković, T., Memišević, V., Bonato, A., Pržulj, N.: Dominating biological networks. PloS One **6**(8), 23016 (2011)
4. Wuchty, S., Stadler, P.F.: Centers of complex networks. Journal of Theoretical Biology **223**(1), 45–53 (2003)
